# Ustekinumab-induced autoimmune hepatitis: a case report

**DOI:** 10.11604/pamj.2023.44.44.38646

**Published:** 2023-01-24

**Authors:** Ali Said Samah, Tennenbaum Ruth, Kallel Roua, Vitte René Louis

**Affiliations:** 1 Service d'Hépato-Gastro-Entérologie, Centre Hospitalier Intercommunal de Poissy, Saint Germain-en-Laye, Yvelines, France

**Keywords:** Ustekinumab, autoimmune hepatitis, drug-induced hepatitis, case report

## Abstract

Ustekinumab is a fully human monoclonal antibody that binds to interleukin (IL)-12/23. Ustekinumab-related liver injury (UST) is rare. There is limited data available on the potential for ustekinumab-liver interaction. We report the case of a patient from our institution followed for colitis ulcerative who developed autoimmune hepatitis (AIH) during treatment with ustekinumab. The diagnosis of autoimmune hepatitis was retained on the simplified criteria for autoimmune hepatitis. Therapeutic management consisted of stopping ustekinumab and starting corticosteroids and immunosuppressants, with regression of cytolysis at 2 months. The purpose of the article is to alert readers and encourage them to report similar cases for better knowledge of the drug.

## Introduction

One-third of patients undergoing biotherapy present a disturbance of the liver balance [1]. A few cases of Autoimmune Hepatitis (AIH) have been reported with INFLIXIMAB, ADALIMUMAB and ETANERCEPT [1,2]. Ustekinumab (UST)-related liver injury is rare. In clinical practice, several recent series of patients treated with USK have not shown any adverse effects on the liver [3]. However, a Spanish observational, retrospective study conducted in 2015 in patients with psoriasis patients receiving ustekinumab showed an increase in grade 1 transaminases in 14% of patients in 14% of patients, although no cases of severe transaminase elevation were observed [4]. On this basis, we feel it is worthwhile to report the case of a patient at our institution who developed AIH during treatment with ustekinumab.

## Patient and observation

**Patient information:** the clinical case we are studying is a 53-year-old patient with no particular history followed for colitis ulcerative since 2013, which became pancolitis in 2018. This patient consulted the hospital on 10/11/21 for acute cytolysis following treatment with ustekinumab.

**Clinical result:** on admission, clinically, the patient was conscious, normocardic at 71 beats/minute and eupneic at 17 beats/minute, afebrile at 37°C. His body mass index was 23 kg/m^2^. The jaundice was frank. Abdominal examination was normal. The rest of the exam is unremarkable.

**Patient history:** his disease was controlled by 5ASA until 2018. Moderate relapse in 04/2018 with a pancolitis lesion extension and simple anal fistula operation. Since then, he has been treated with AZATHIOPRINE without efficacy for one year and switched to ADALIMUMAB in 04/2019. Psoriasiform dermatosis motivated the switch to GOLIMUMAB in 03/2020 with a therapeutic escape. Switch to UST in 08/2021. First infusion on 03/08/2021 of 520 mg and subcutaneous injection of 90 mg on 02/10/2021. The evolution was marked by the appearance of acute hepatitis due to the introduction of ustekinumab.

**Figure 1:**
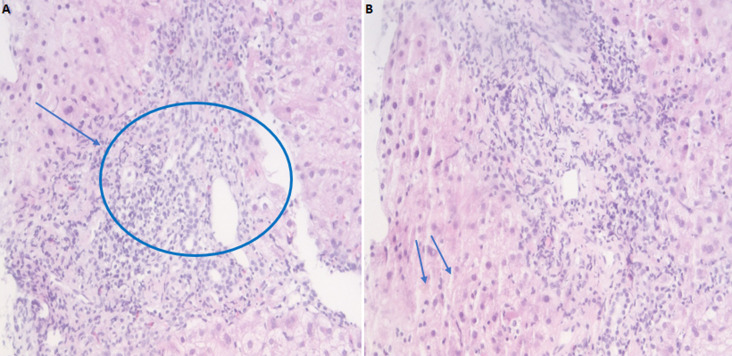
A) polymorphic infiltrate essentially lymphoplasmacytic moderate portal with the presence of a few rare eosinophilic polynuclears; B) lesions of patchy necrosis

**Diagnostic approach:** the patient underwent a biological assessment showing a biological from 30/08 with a maximum aspartate aminotransferase (AST) 254 (normal: 40 UI/L), ALT 548 (normal: 40 UI/l), GGT 106 (normal: 80 UI/l), PAL normal. Etiological work-up; minimal alcohol consumption at 3 g/day and none from 10/2021. Body Mass Index (BMI)=27.8 kg/m^2^. Abdominal ultrasound: hepatic steatosis, no biliary dilatation. BILI Magnetic resonance imaging: no evidence of cholangitis. Serologies, Hepatitis B Virus (HBV), Hepatitis C Virus (HCV), Human Immunodeficiency Virus (HIV), Epstein-Barr Virus (EBV), Cytomegalovirus (CMV) IgM negative, Hepatitis E Virus (HEV) IgG positive, HEV IgM and Ribonucleic Acid (RNA) negative. Copper test negative AAN=1/1280, anti-smooth muscle=1/80, anti LkM1, anti LC1 negative Gammaglobulins=16 g/dl. Liver biopsy showed chronic inflammatory non-specific hepatitis with polymorphic inflammatory hepatitis with a polymorphic infiltrate of focal piecemeal, possibly autoimmune F1 F2 without cholangitis (Figure 1). The pre-treatment liver function test was strictly normal. When the patient's history was reviewed, an episode of cytolysis was described in 2016 at 1.5 N spontaneously resolved, ANA=1/640, anti-M smooth negative, anti-Neutrophil Cytoplasmic antibody (ANCA) positive, no signs of cholangitis on MRI. In 2021, the diagnosis of USTEKINIMAB-induced autoimmune hepatitis (AIH) was retained due to the time of onset, antibody positivity and histological data with a Hepatitis Activity Index (HAI) score of 7.

**Therapeutic intervention and follow-up:** treatment was suspended and replaced by ENTYVIO (vedolizumab) and corticosteroids with a rapid improvement of the liver function rapid improvement of liver function on PREDNISONE. The rebound of cytolysis following discontinuation of on 25/04/2022 AST/ALT=127/319 indication to put back on corticosteroids and azathioprine with weekly monitoring of liver function tests, complete blood count (CBC) for one month then once a month for three months for 3 months and then every 3 months. Spontaneous regression of cytolysis was noted after 2 months of treatment with biology: ASAT 32 (N). ALT 47 (N < 40). GGT 41. PAL 62, total bilirubin 15μmol/l (N).

## Discussion

Contrary to other biotherapies, UST is rarely incriminated in autoimmune manifestations paradoxical manifestations. It has been described as safe from a hepatic point of view according to phase III and IV studies and PHOENIX I and II studies in dermatology. In the PHOENIX I and II studies, normal transaminase values In PHOENIX I and II, normal transaminase values were always recorded in enrolled patients [5]. A similar case was reported by Lovero R in 2018, it was a major cytolysis following UST infusion [6]. The etiological investigation was negative. The liver biopsy showed a lymphocytic infiltration, cholangitis and piecemeal necrosis. The diagnosis retained was that of treatment-induced liver damage without HAI [6]. Spontaneous improvement of liver function after 6 months of treatment was observed without recourse to corticosteroids and without recurrence, contrary to our case [6]. For our patient, was the UST the cause of a decompensation of an unrecognized AIH in the face of a history of positive NAA (nucleic acid amplification)? Drug-induced autoimmune hepatitis is an almost complete mimicry of classic autoimmune hepatitis [7]. Relapse after corticosteroid withdrawal is an important distinction between classical autoimmune hepatitis and drug-induced autoimmune hepatitis [7]. Our patient relapsed on discontinuation of corticosteroid therapy. In classical autoimmune hepatitis, the immunological disturbances persist and their persistence implies that the triggering antigen is constant or that the critical protective immune regulatory mechanisms are permanently impaired [8].

Mechanisms are permanently impaired [8]. Autoimmune hepatitis can specifically occur in various forms: inflammatory bowel disease, including solitary autoimmune hepatitis, overlap with primary biliary cholangitis or primary sclerosing cholangitis, or as drug-induced autoimmune hepatitis induced by drugs due to the treatment of inflammatory bowel disease (IBD) [9]. In our case, the liver enzymes were normal before the introduction of ustekinumab, which is consistent with ustekinumab-induced autoimmune hepatitis. Immune-induced hepatitis. In our case, we can consider that ustekinumab was the trigger of an ustekinumab was the trigger for an autoimmune hepatitis that was probably latent until now. The diagnosis of AIH in our patient was based on clinical, laboratory and histological findings. Histological findings. The simplified score was calculated to be 7, so it is certainly AIH (Table 1). The absence of other causal factors strongly suggests that this patient presented with an autoimmune hepatitis urekinumab-induced autoimmune hepatitis.

**Table 1:** simplified autoimmune hepatitis score

Auto-antibodies AAN=1/1280	>1: 80 = 2 points	2 points
IgG 16 g/dl	Above normal limit	1 points
Histology	Typical of autoimmune hepatitis	2 points
Absence of viral hepatitis	Yes	2 points
Total score		7 points: HAI certain

Ustekinumab is a neutralizing monoclonal antibody that recognizes the p40 protein. This protein is a common subunit of IL-12 and IL-23 and thus prevents these cytokines from binding to the surface receptor of the naive lymphocyte. It blocks the differentiation and multiplication of TH1 and TH17 lymphocytes and thus reduces the production of proinflammatory cytokines [10]. Recent findings highlight the role of the interaction between CD4 cells, CD25, regulatory T cells and Th17 cells in the pathogenesis of autoimmune hepatitis. An imbalance between regulatory and effector cells in favor of the latter seems to be a determining factor in the in favor of the latter seems to be a determining factor in the progression of the disease. In addition, the intrahepatic microenvironment of autoimmune hepatitis is particularly rich in pro-inflammatory cytokines such as: IL-6, IL-17, IL-23, IL-1b which play a crucial role in the perpetuation and expansion of effector cells and subsequent damage to the effector cells and subsequent liver damage, while regulatory T cells are greatly disadvantaged and inhibited in such a polarized habitat [11]. Th1 and Th17 cells are involved in the pathogenesis of AIH, so a favorable impact of ustekinumab on effectors of liver injury is expected [11]. But the question remains to understand how it can induce autoimmune hepatitis. This pathophysiological mechanism is not well understood. Some of the mechanisms proposed for the induction of autoimmune hepatitis include underlying mechanisms of genetic susceptibility, a selective effect of T-helper cells, formation of immune complexes, and induction of an immune system imbalance related to cytokine blockade [12]. As detailed above, drug-induced hepatitis in relation to UST has been reported previously, however, our case is the first reported in the literature of an autoimmune hepatitis induced by UST.

## Conclusion

The result of the case study suggests the possibility that ustekinumab may induce autoimmune hepatitis. This was resolved by the withdrawal of the drug and initiation of corticosteroid therapy and immunosuppressant. On this basis, although a direct relationship between the administration of the drug and the occurrence of the observed event cannot be established beyond doubt, our report is intended to alert readers and to encourage them to report similar cases for better knowledge.
